# Frequency of Neurological Diseases After COVID-19, Influenza A/B and Bacterial Pneumonia

**DOI:** 10.3389/fneur.2022.904796

**Published:** 2022-06-23

**Authors:** Pardis Zarifkar, Costanza Peinkhofer, Michael E. Benros, Daniel Kondziella

**Affiliations:** ^1^Department of Neurology, Rigshospitalet, Copenhagen University Hospital, Copenhagen, Denmark; ^2^Copenhagen Research Center for Mental Health–CORE, Mental Health Center Copenhagen, Copenhagen University Hospital, Copenhagen, Denmark; ^3^Department of Immunology and Microbiology, University of Copenhagen, Copenhagen, Denmark; ^4^Department of Clinical Medicine, University of Copenhagen, Copenhagen, Denmark

**Keywords:** COVID-19, SARS-CoV-2, bacterial pneumonia, Alzheimer's disease (AD), Parkinson's disease (PD), ischemic stroke (IS), auto-immune

## Abstract

**Introduction:**

COVID-19 might affect the incidence of specific neurological diseases, but it is unknown if this differs from the risk following other infections. Here, we characterized the frequency of neurodegenerative, cerebrovascular, and immune-mediated neurological diseases after COVID-19 compared to individuals without COVID-19 and those with other respiratory tract infections.

**Methods:**

This population-based cohort study utilized electronic health records covering ~50% of Denmark's population (*n* = 2,972,192). Between 02/2020 and 11/2021, we included individuals tested for COVID-19 or diagnosed with community-acquired bacterial pneumonia in hospital-based facilities. Additionally, we included individuals tested for influenza in the corresponding pre-pandemic period between 02/ 2018 and 11/2019. We stratified cohorts for in- and outpatient status, age, sex, and comorbidities.

**Results:**

In total, 919,731 individuals were tested for COVID-19, of whom 43,375 tested positive (35,362 outpatients, 8,013 inpatients). Compared to COVID-negative outpatients, COVID-19 positive outpatients had an increased RR of Alzheimer's disease (RR = 3.5; 95%CI: 2.2–5.5) and Parkinson's disease (RR = 2.6; 95%CI: 1.7–4.0), ischemic stroke (RR = 2.7; 95%CI: 2.3–3.2) and intracerebral hemorrhage (RR = 4.8; 95%CI: 1.8–12.9). However, when comparing to other respiratory tract infections, only the RR for ischemic stroke was increased among inpatients with COVID-19 when comparing to inpatients with influenza (RR = 1.7; 95%CI: 1.2–2.4) and only for those >80 years of age when comparing to inpatients with bacterial pneumonia (RR = 2.7; 95%CI: 1.2–6.2). Frequencies of multiple sclerosis, myasthenia gravis, Guillain-Barré syndrome and narcolepsy did not differ after COVID-19, influenza and bacterial pneumonia.

**Conclusion:**

The risk of neurodegenerative and cerebrovascular, but not neuroimmune, disorders was increased among COVID-19 positive outpatients compared to COVID-negative outpatients. However, except for ischemic stroke, most neurological disorders were not more frequent after COVID-19 than after other respiratory infections.

## Introduction

Neurological symptoms, including headache and anosmia, are present in more than 80% of hospitalized COVID-19 patients (1, 2). There is also evidence of an inflammatory hypercoagulable state with subsequent cerebrovascular incidents, (3–8) and case descriptions exist of Guillain-Barré syndrome (GBS) and Parkinson's disease following COVID-19 (9, 10). To our knowledge, however, epidemiologic studies investigating the incidence of specific neurodegenerative diseases such as Alzheimer's disease and Parkinson's disease or auto-immune disorders (e.g., multiple sclerosis, narcolepsy, and myasthenia gravis) after COVID-19 are still missing.

The aim of this study was to provide the first broad investigation into the influence of COVID-19 on neurological diseases, providing a rapid glimpse based on the electronic health record data currently available while awaiting more detailed longitudinal nationwide registry studies. Specifically, we aimed to (1) characterize the frequency and relative risk (RR) of neurodegenerative, cerebrovascular, and immune-mediated diseases in patients with COVID-19, and (2) to compare the risk of being diagnosed with a neurological disease after COVID-19 to the risk after influenza A/B and community-acquired bacterial pneumonia.

## Methods

### Study Population

Using previously published methods, (5) we extracted patient data from electronic health records covering 2,972,192 individuals, equating to ~50% of the Danish population from two (of five in total) well-defined administrative regions in Denmark, i.e., the Capital Region (Greater Copenhagen and Bornholm) and Region Zealand. The electronic health records (EPIC, version 2021, Wisconsin, USA) Slicer-Dicer function, were searched from implementation in 2016 to November 27, 2021. All individuals ≥ 18 years who were tested in a hospital setting for COVID-19, influenza A/B (referred to as influenza) or diagnosed with community-acquired bacterial pneumonia (referred to as bacterial pneumonia) were followed for new-onset neurological diseases up to 12 months later. Included individuals were (1) hospitalized patients tested for COVID-19, influenza, or diagnosed with bacterial pneumonia during admission (referred to as “inpatients”), and (2) non-hospitalized patients tested during ambulatory visits, or healthy individuals tested in hospital-based facilities that serve the general population (referred to as “outpatients”). Individuals tested for COVID-19 in the community setting (e.g., over-the-counter antigen tests or PCR tests from private providers and primary care settings) were not captured. We also collected anonymized aggregated data on age, sex, smoking, pre-existing comorbidities, laboratory data, medical prescriptions, and history of neurological disorders. Data extraction and analysis were conducted in consultation with EPIC data experts from our institution (Rigshospitalet, Copenhagen University Hospital) according to previous publications by our group (5).

Slicer Dicer search strategies are detailed in Supplementary Table 1.

### Study Period

The study period spanned from February 27, 2018 to November 27, 2021. COVID-19 and bacterial pneumonia patients were included from February 27, 2020 (the first reported case of COVID-19 in Denmark) (11) to November 27, 2021 (the day before the first reported case of the omicron variant in Denmark) (12), and influenza patients from February 27, 2018, to November 27, 2019 (the corresponding 2-year pre-pandemic period).

### Assessment of Infection Exposure

COVID-19 or influenza positive cases were determined by positive reverse-transcriptase polymerase chain reaction assays of nasal, pharyngeal, or tracheal samples. We defined COVID-19 or influenza negative cases as having negative laboratory test results and (for those tested more than once) no previous history of positive laboratory tests.

### Assessment of Neurological Outcomes

Using ICD-10 diagnoses, we identified individuals with neurodegenerative (Alzheimer's disease, Parkinson's disease), cerebrovascular (ischemic stroke, intracerebral hemorrhage, subarachnoid hemorrhage), and immune-mediated (multiple sclerosis, GBS, myasthenia gravis, and narcolepsy) disorders. ICD-10 diagnosis codes are detailed in Supplementary Table 1.

### Statistical Analyses

We calculated the risk of new-onset neurological diagnoses in the acute (1 month), subacute (3 and 6 months), and chronic (12 months) phases after a diagnosis of COVID-19, influenza, or bacterial pneumonia. Specifically, we calculated the relative risk (RR) of diagnosis rates with 95% confidence intervals (CI) and stratified the study population across admittance status (inpatients and outpatients), age (18–39, 40–59, 60–79, and ≥80 years), and sex (male and female), using R studio (2021 Vienna, Austria). To reduce the risk of type II errors, statistical analyses were only conducted for diseases with ≥4 cases in each group. Hospitalization and delirium [which occurs at higher rates in COVID-19 patients (Table 1)] can lead to cognitive decline and aggravate neurodegenerative diseases (13–16). Thus, to best balance recovery from hospitalization and allow for reliable diagnoses, Alzheimer's disease and Parkinson's disease patients diagnosed within the first 3 months after admission were excluded from 6 and 12-month assessments (14–16).

**Table 1:** Clinical characteristics and demographics at baseline.

	**Inpatient status at baseline**				**Outpatient status at baseline**		
	**COVID-19 positive** **(*n* = 8,013)**	**COVID-19 negative** **(*n* = 230,686)**	**Influenza positive** **(*n* = 4,142)**	**Pneumonia** **(*n* = 1,474)**	**COVID-19 positive** **(*n* = 35,362)**	**COVID-19 negative** **(*n* = 645,670)**	**Influenza positive** **(*n* = 3,960)**
**Age**, ***n*** **(%)**							
Mean, years	66y	58y	68y	75y	48y	47y	52y
18–39	1,023 (12.8%)	65,333 (28%)	508 (12.3%)	44 (3%)	14,309 (40.1%)	258,412 (40%)	1,352 (34.1%)
40–59	1,841 (23%)	38,108 (20.9%)	854 (20.6%)	140 (9.5%)	12,526 (35.4%)	234,480 (36.3%)	1,482 (37.4%)
60–79	3,128 (39%)	76,865 (33.3%)	1,743 (42.08%)	671 (45.5%)	5,731 (16.2%)	128,382 (19.9%)	929 (23.4%)
≥80	2,021 (25.2%)	40,380 (17.5%)	1,037 (25%)	619 (42%)	2,796 (7.9%)	24,396 (3.8%)	197 (5%)
**Sex**, ***n*** **(%)**							
Females	3,567 (44.5%)	131,399 (57%)	2,257 (54%)	625 (42.4%)	20,913 (59.1%)	368,142 (57%)	2,374 (59.9%)
**Smoking status**, ***n*** **(%)**							
Current or history of smoking (%)	3,141 (39.2%)	93,283 (40.4%)	2,053 (49.6%)	829 (56.2%)	6,180 (17.5%)	117,505 (18.2%)	931 (23.5%)
**Pre-existing comorbidities**, ***n*** **(%)**							
Celiac disease	11 (0.1%)	370 (0.2%)	1 (0.02%)	1 (0.07%)	51 (0.1%)	1,071 (0.2%)	3 (0.08%)
Delirium	149 (1.9%)	1,335 (1%)	19 (0.5%)	33 (2.2%)	127 (0.4%)	429 (0.1%)	1 (0.03%)
Diabetes mellitus, type 1	30 (0.4%)	719 (0.3%)	16 (0.4%)	7 (0.5%)	66 (0.2%)	974 (0.2%)	9 (0.2%)
Diabetes mellitus, type 2	501 (6.2%)	7,880 (3.4%)	142 (3.4%)	102 (6.9%)	4,397 (1.4%)	5,335 (0.8%)	30 (0.8%)
Hashimoto's auto-immune thyroiditis	11 (0.14%)	417 (0.2%)	5 (0.1%)	0 (0%)	67 (0.2%)	1,103 (0.2%)	3 (0.08%)
Hypercholesterolemia	431 (5.4%)	9,571 (4.2%)	118 (2.9%)	83 (5.6 %)	560 (1.6%)	8,303 (1.3%)	39 (1%)
Hypertension	1,681 (21%)	36,754 (15.9%)	519 (12.5%)	411 (27.9%)	2,155 (6.1%)	29,935 (4.6%)	155 (4%)
Ischemic stroke	340 (4.2%)	10,030 (4.4%)	53 (1.3%)	96 (6.5%)	442 (1.3%)	3,829 (0.6%)	26 (0.7%)
Obesity	356 (4.4%)	10,962 (4.8%)	60 (1.5%)	36 (2.4%)	959 (2.7%)	15,465 (2.4%)	56 (1.4%)
Rheumatoid arthritis	46 (0.6%)	982 (0.4%)	22 (0.5%)	13 (0.9%)	66 (0.2%)	995 (0.2%)	10 (0.2%)
Transitory cerebral ischemia	130 (1.6%)	4,197 (1.8%)	24 (0.6%)	37 (2.5%)	220 (0.6%)	2,505 (0.4%)	7 (0.2%)

### Sensitivity Analyses

To search for possible bias related to restricted access to diagnostic work-up during the pandemic, the prevalence of disease-specific diagnostic procedures (including cerebral fluorodeoxyglucose (FDG)- positron emission tomography (PET)-18 for Alzheimer's disease and single-photon emission computerized tomography (SPECT) for Parkinson's disease), medical prescriptions and common risk factors, including smoking status, and pre-existing comorbidities were compared across groups using chi-squared statistics with a Yates correction. Where there was a significant difference in risk factors between groups, the populations at risk were excluded from comparative analyses.

### Ethics and Data Availability Statement

The Scientific Ethics Committee of the Capital Region of Denmark waives approval for register-based studies on aggregated anonymized data (Section 14.2, Committee Act 2). The datasets included in this study are freely available to medical and administrative staff in Denmark with access to electronic health records in EPIC.

## Results

**Figure 1:**
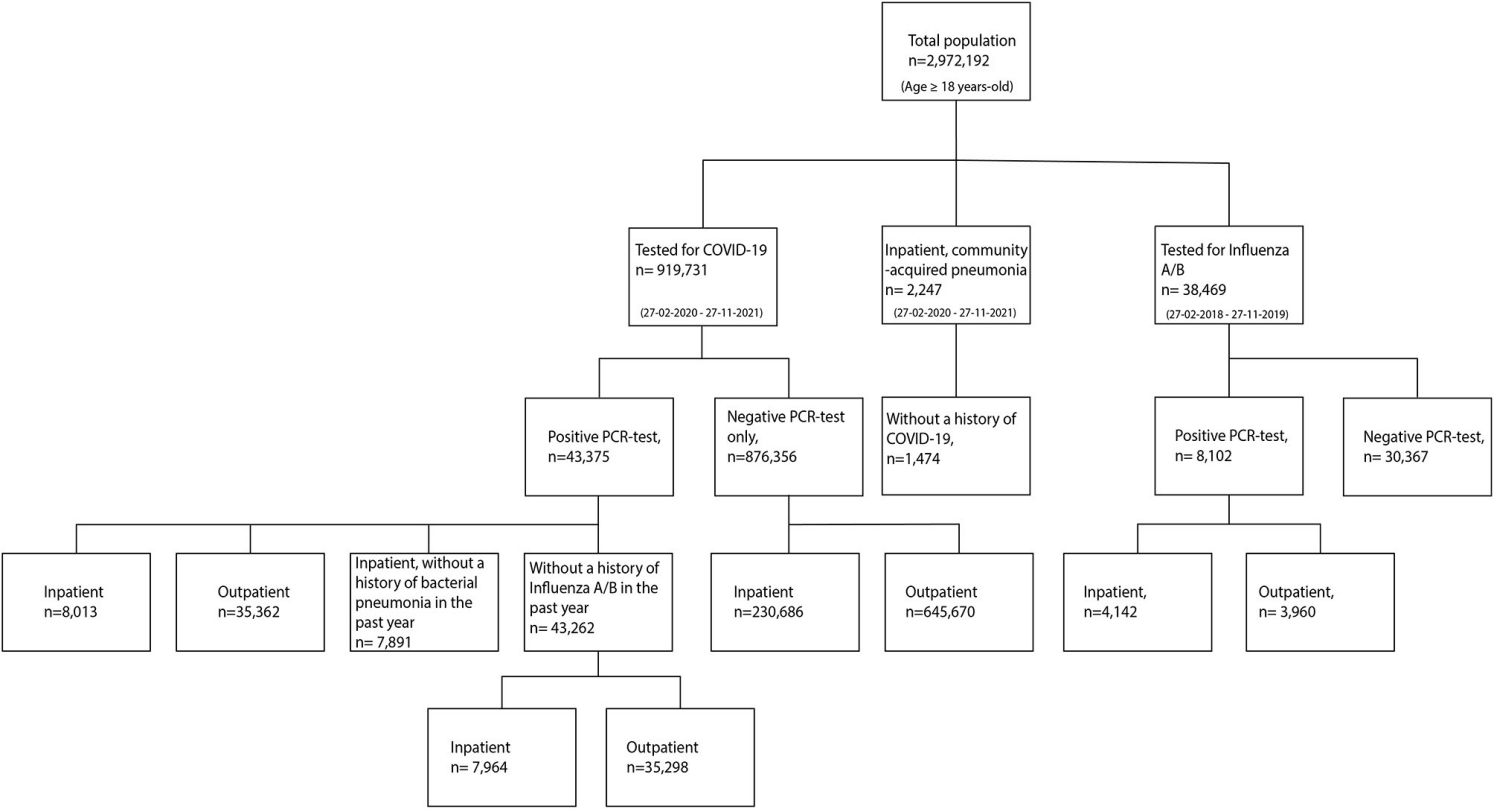
Flowchart of individuals tested for COVID-19 or Influenza A/B, and diagnosed with community-acquired bacterial pneumonia.

Between February 27, 2020 and November 27, 2021, a total of 919,731 individuals were tested for COVID-19 in a hospital-based facility. Of these, 43,375 individuals had a positive COVID-19 test (equating to 20% of the COVID-19 positive population in the surveyed areas) (17) and 876,356 had a negative COVID-19 test (40% of the COVID-negative population in these areas) (18). A total of 1,474 individuals were diagnosed with bacterial pneumonia in a hospital-based facility during the same period. Between February 27, 2018 and November 27, 2019, a total of 8,102 individuals were tested positive for influenza. A flowchart of the study population is depicted in Figure 1, and demographic and clinical characteristics are detailed in Table 1.

### Risk Factors at Baseline

The prevalence and comparative analyses of clinical baseline characteristics are detailed in Table 1 and Supplementary Table 2. Compared to COVID-negative individuals (in- and outpatients separately and combined) and influenza inpatients, COVID-19 positive individuals carried higher rates of some pre-existing cerebrovascular risk factors, (19) including hypercholesterolemia, diabetes mellitus type 2 and hypertension. Compared to COVID-negative outpatients and influenza inpatients, COVID-19 positive individuals also had higher rates of obesity, and a history of transitory ischemic attack. By contrast, smoking rates were higher among COVID-negative individuals, and influenza and pneumonia inpatients. Pneumonia inpatients also had higher rates of past transitory ischemic attacks. There were no other differences in cerebrovascular risk factors, nor in the rates of pre-existing auto-immune disorders.

### The Incidence of New-Onset Neurodegenerative, Cerebrovascular and Auto-Immune Disorders

**Figure 2:**
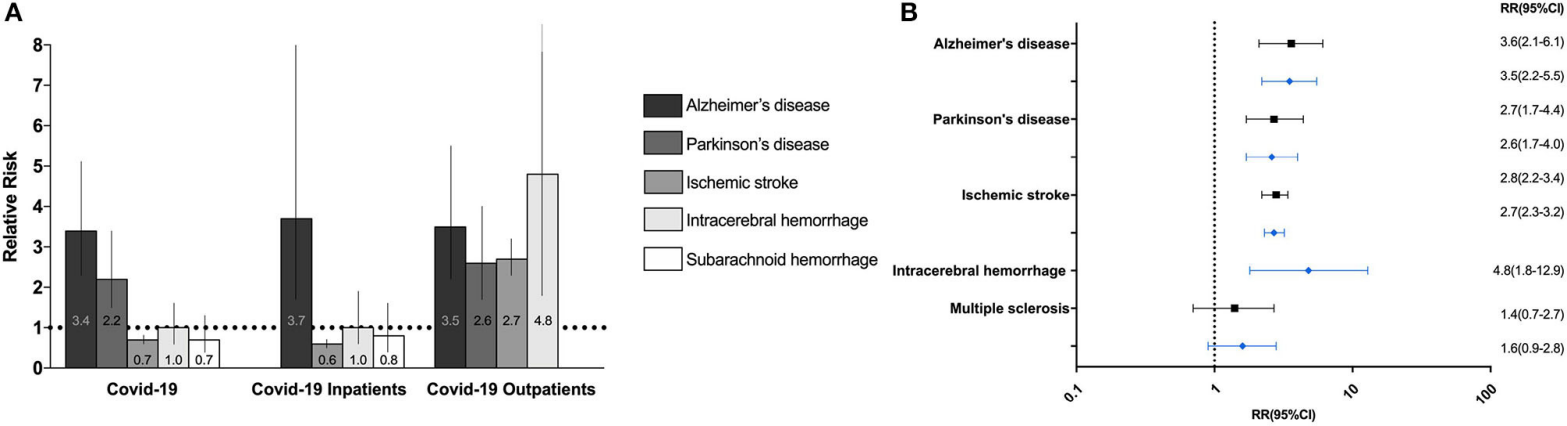
Relative risk of neurodegenerative, cerebrovascular and neuroimmune neurological disorders after COVID-19 **(A)**. Bar chart of the relative risks (RR) of new-onset neurodegenerative disorders and cerebrovascular events after 12 months in COVID-19 positive versus COVID-negative individuals, inpatients, and outpatients. Barcharts depict RR with 95% confidence intervals. **(B)** Forest plot of the RR of new-onset neurodegenerative, cerebrovascular and neuroimmune disorders six (black) and twelve (blue) after COVID-19 in COVID-19 positive outpatients compared to negative outpatients.

The incidence, absolute risk, and RR of all neurological diseases in COVID-19 positive and COVID-negative individuals are depicted in Figure 2 and Table 2. Stratifications by age and sex are detailed in Supplementary Table 3, and stratifications by in- and outpatient status are detailed in Supplementary Table 4.

**Table 2:** Relative risk of neurodegenerative, cerebrovascular and neuroimmune disorders in COVID-19 positive compared to COVID-negative individuals.

	**COVID-19 positive** **(*n* = 43,375)**	**COVID-19 negative** **(*n* = 876,356)**	**RR (95%CI)**	**COVID-19 positive** **(*n* = 43,375)**	**COVID-19 negative** **(*n* = 876,356)**	**RR (95%CI)**	**COVID-19 positive** **(*n* = 43,375)**	**COVID-19 negative** **(*n* = 876,356)**	**RR (95%CI)**	**COVID-19 positive** **(*n* = 43,375)**	**COVID-19 negative** **(*n* = 876,356)**	**RR** **(95%CI)**
	**1 month**, ***n*** **(%)**			**3 months**, ***n*** **(%)**			**6 months**, ***n*** **(%)**			**12 months**, ***n*** **(%)**		
Alzheimer's disease^♦^	-	-	-	-	-	-	21 (0.05%)	121 (0.01%)	**3.5** (2.2–5.6)*	29 (0.07%)	171 (0.02%)	**3.4** (2.3–5.1)*
Parkinson's disease^♦^	-	-	-	-	-	-	20 (0.05%)	169 (0.02%)	**2.4** (1.5–3.8)*	26 (0.06%)	234 (0.03%)	**2.2** (1.5–3.4)*
Ischemic stroke	117 (0.3%)	6,251 (0.7%)	**0.4** (0.3–0.5)*	180 (0.4%)	6,908 (0.8%)	**0.6** (0.5–0.7)*	227 (0.5%)	7,365 (0.8%)	**0.6** (0.5–0.7)*	281 (0.6%)	7,910 (0.9%)	0.7 (0.6–0.8)
Intracerebral hemorrhage	7 (0.02%)	250 (0.03%)	0.6 (0.3–1.2)	10 (0.02%)	280 (0.03%)	0.7 (0.4–1.4)	13 (0.03%)	306 (0.03%)	0.9 (0.5–1.5)	16 (0.04%)	330 (0.04%)	1.0 (0.6–1.6)
Subarachnoid hemorrhage	4 (0.01%)	201 (0.02%)	0.4 (0.1–1.1)	5 (0.01%)	233 (0.03%)	0.4 (0.2–1.1)	6 (0.01%)	254 (0.03%)	0.5 (0.2–1.1)	ll10 (0.02%)	289 (0.03%)	0.7 (0.4–1.3)
Guillain-Barré syndrome	1 (0.002%)	52 (0.006%)	N/A	2 (0.005%)	58 (0.007)	N/A	2 (0.005)	61 (0.007%)	N/A	2 (0.005)	64 (0.007%)	N/A
Multiple sclerosis	4 (0.01%)	185 (0.02%)	0.4 (0.2–1.2)	6 (0.01%)	246 (0.03%)	0.5 (0.2–1.1)	11 (0.03%)	293 (0.03%)	0.8 (0.4–1.4)	14 (0.03%)	332 (0.04%)	0.9 (0.5–1.5)
Myasthenia gravis	1 (0.002%)	44 (0.005%)	N/A	1 (0.002%)	59 (0.007%)	N/A	1 (0.002%)	61 (0.007%)	N/A	1 (0,002%)	71 (0.008%)	N/A
Narcolepsy	0 (0.0%)	19 (0.002%)	N/A	0 (0.0%)	30 (0.003%)	N/A	0 (0.0%)	37 (0.004%)	N/A	0 (0.0%)	41 (0.005%)	N/A

*Statistical analyses were only conducted for diseases with ≥ 4 cases in each group*.

* *Statistically significant RR (p < 0.05) are highlighted in bold*.

*^♦^Excluding inpatient cases of Alzheimer's disease and Parkinson's disease the first three months after hospitalization with COVID-19*.

The incidences, absolute risks, and RR's of all neurological diseases in COVID-19 positive, influenza positive, and bacterial pneumonia patients are depicted in Table 3 and Supplementary Table 5.

**Table 3:** Relative risk of neurodegenerative, cerebrovascular and neuroimmune disorders in inpatients with COVID-19 compared to influenza inpatients and community-acquired bacterial pneumonia inpatients.

	**COVID- 19 positive** **(*n* = 7,964)**	**Influenza positive** **(*n* = 4,142)**	**RR (95%CI)**	**COVID-19 positive** **(*n* = 7,964)**	**Influenza positive** **(*n* = 4,142)**	**RR (95%CI)**	**COVID-19 positive** **(*n* = 7,891)**	**Pneumonia** **(*n* = 1,474)**	**RR (95%CI)**	**COVID-19 positive** **(*n* = 7,891)**	**Pneumonia** **(*n* = 1,474)**	**RR** **(95%CI)**
	**1 month**, ***n*** **(%)**			**3 months**, ***n*** **(%)**			**1 month**, ***n*** **(%)**			**3 months**, ***n*** **(%)**		
Ischemic stroke	85 (1.07%)	26 (0.63%)	**1.7** (1.1–2.6)*	113 (1.4%)	34 (0.8%)	**1.7** (1.2–2.5)*	79 (1.0%)	14 (0.9%)	1.1 (0.6–1.9)	107 (1.4%)	19 (1.3%)	1.18 (0.6–1.7)
Intracerebral hemorrhage	6 (0.08%)	0 (0.0%)	N/A	8 (0.1%)	0 (0.0%)	N/A	9 (0.1%)	0 (0.0%)	N/A	8 (0.1%)	0 (0.0%)	N/A
Subarachnoid hemorrhage	4 (0.05%)	0 (0.0%)	N/A	5 (0.06%)	0 (0.0%)	N/A	6 (0.08%)	0 (0.0%)	N/A	5 (0.06%)	0 (0.0%)	N/A
Guillain-Barré syndrome	1 (0.01%)	2 (0.05%)	N/A	1 (0.01%)	2 (0.05%)	N/A	1 (0.01%)	1 (0.07%)	N/A	1 (0.01%)	1 (0.07%)	N/A
Multiple sclerosis	1 (0.01%)	0 (0.0%)	N/A	1 (0.01%)	1 (0.02%)	N/A	1 (0.01%)	2 (0.1%)	N/A	1 (0.01%)	2 (0.1%)	N/A
Myasthenia gravis	1 (0.01%)	0 (0.0%)	N/A	1 (0.01%)	0 (0.0%)	N/A	1 (0.01%)	0 (0.0%)	N/A	1 (0.01%)	0 (0.0%)	N/A
Narcolepsy	0 (0.0%)	0 (0.0%)	N/A	0 (0.0%)	0 (0.0%)	N/A	0 (0.0%)	0 (0.0%)	N/A	0 (0.0%)	0 (0.0%)	N/A
	**6 months**, ***n*** **(%)**			**12 months**, ***n*** **(%)**			**6 months**, ***n*** **(%)**			**12 months**, ***n*** **(%)**		
Alzheimer's disease	4 (0.05%)	1 (0.02%)	N/A	7 (0.09%)	3 (0.07%)	N/A	4 (0.05%)	0	N/A	7 (0.09%)	0 (0.0%)	N/A
Parkinson's disease	0 (0.0%)	0 (0.0%)	N/A	2 (0.03%)	4 (0.1%)	N/A	1 (0.01%)	0	N/A	3 (0.04%)	3 (0.2%)	N/A
Ischemic stroke	128 (1.6%)	39 (0.9%)	**1.7** (1.2–2.4)*	145 (1.8%)	58 (1.4%)	1.3 (1.0–1.8)	121 (1.5%)	23 (1.6%)	1.0 (0.6–1.5)	139 (1.8%)	28 (1.9%)	0.9 (0.6–1.4)
Intracerebral hemorrhage	10 (0.1%)	0 (0.0%)	N/A	11 (0.14%)	1 (0.02%)	N/A	10 (0.1%)	0 (0.0%)	N/A	11 (0.1%)	0 (0.0%)	N/A
Subarachnoid hemorrhage	5 (0.06%)	0 (0.0%)	N/A	7 (0.09%)	0	N/A	5 (0.1%)	0 (0.0%)	N/A	7 (0.1%)	0 (0.0%)	N/A
Guillain-Barré syndrome	1 (0.01%)	2 (0.05%)	N/A	1 (0.01%)	2 (0.05%)	N/A	1 (0.01%)	1 (0.07%)	N/A	1 (0.01%)	1 (0.07%)	N/A
Multiple sclerosis	1 (0.01%)	2 (0.05%)	N/A	1 (0.01%)	2 (0.05%)	N/A	1 (0.01%)	2 (0.1%)	N/A	1 (0.01%)	2 (0.1%)	N/A
Myasthenia gravis	1 (0.01%)	0 (0.0%)	N/A	1 (0.01%)	0 (0.0%)	N/A	1 (0.01%)	0 (0.0%)	N/A	1 (0.01%)	0 (0.0%)	N/A
Narcolepsy	0 (0.0%)	0 (0.0%)	N/A	0 (0.0%)	0 (0.0%)	N/A	0 (0.0%)	0 (0.0%)	N/A	0 (0.0%)	0 (0.0%)	N/A

*Statistical analyses were only conducted for diseases with ≥ 4 cases in each group*.

* *Statistically significant RR (p < 0.05) are highlighted in bold*.

### Alzheimer's Disease and Parkinson's Disease

The RR of Alzheimer's disease was increased 6 and 12 months after a positive test in COVID-19 positive compared to COVID-negative individuals (in- and outpatients combined), and separately among in- and outpatients (in- and outpatients: RR = 3.5; 95%CI: 2.5–5.6 at 6 months and RR = 3.4; 95%CI: 2.3–5.1 at 12 months; inpatients: six (RR = 3.3; 95%CI: 1.7–9.3 at 6 months and RR = 3.7; 95%CI: 1.7–8.0 at 12 months; outpatients; RR = 3.6; 95%CI: 2.1–6.1 at 6 months and RR = 3.5, 95%CI: 2.2–5.5 at 12 months).

Notably, COVID-19 positive individuals had a higher frequency of delirium, an independent risk factor for dementia (20) (0.6 vs. 0.3%, χ^2^ = 128.2, *p* < 0.00001), compared to COVID-negative individuals. After exclusion of those with a history of delirium, the RR for Alzheimer's disease remained elevated in COVID-19 individuals (in- and outpatients combined) (Supplementary Table 6), and separately in both in- and outpatients. COVID-19 positive individuals also had a higher frequency of cerebrovascular risk factors (Table 1 and Supplementary Table 2). After exclusion of those with cerebrovascular risk factors, the RR for Alzheimer's disease remained elevated in COVID-19 individuals (Supplementary Table 6). In the inpatient group, there were too few cases for statistical analyses.

The RR of Parkinson's disease was increased 6 and 12 months after a positive test in COVID-19 positive compared to COVID-negative individuals (in- and outpatients combined) and specifically in COVID-19 outpatients (in- and outpatients combined: RR = 2.4; 95%CI: 1.5–3.8 at 6 months and RR = 2.2; 95%CI: 1.5–3.4 at 12 months; outpatients: RR = 2.7; 95%CI: 1.7–4.4 at 6 months and RR = 2.6; 95% CI: 1.7–4.0. Among inpatients, there were not enough Parkinson's disease cases to conduct meaningful statistics. Finally, there was no excess risk of Alzheimer's disease or Parkinson's disease compared to influenza or bacterial pneumonia inpatients (Table 3).

From February 27, 2020 to November 27, 2021, 1,137 cerebral PET-FDG-18 scans were conducted in COVID-19 positive individuals and 23,889 in COVID-negative individuals, corresponding to a 3% scanning rate in each group (χ^2^ = 1.7, *p* = 0.19). Similarly, there was no difference in the number of SPECT scans among COVID-19 positive and negative individuals (0.04 vs. 0.03%, χ^2^ = 2.1, *p* = 0.14), indicating equal access to these diagnostic tools.

### Ischemic Stroke

The frequency of new-onset ischemic stroke did not differ significantly between COVID-19 positive and COVID-negative individuals (in- and outpatients combined), nor between COVID-19 positive and COVID-negative inpatients (Table 2 and Supplementary Table 4). Compared to COVID-negative outpatients, the RR of ischemic stroke was increased three, six, and 12 months after a positive test in COVID-19 positive outpatients but was insignificant within the first month (RR = 1.4, 95%CI: 1.0–2.0, *p* = 0.08 after 1 month, RR = 2.3; 95% CI: 1.8–3.0 after 3 months, RR = 2.8; 95%CI: 2.2–3.4 after 6 months and RR = 2.7; 95%CI: 2.3–3.2 after 12 months). Notably, age-specific stratifications showed that the relative risk was highest among younger patients between 40 and 59 years (Supplementary Table 3). After exclusion of cerebrovascular risk factors, the RR for ischemic stroke remained elevated in COVID-19 positive outpatients (RR = 1.8; 95%CI: 1.5–2.8 after 3 months, RR = 2.2; 95% CI:1.5–3.1 after 6 months, and RR = 2.1; 95% CI:1.5–2.8 after 12 months).

Compared to influenza positive inpatients, COVID-19 inpatients had an increased RR of ischemic stroke one, three, and 6 months after a positive test (RR = 1.7; 95%CI: 1.1–2.6 after 1 month; RR = 1.7; 95%CI: 1.2–2.5 after 3 months; RR = 1.7; 95%CI: 1.2–2.4 after 6 months). After 12 months, the RR between the two groups was decreased (RR = 1.3; 95%CI: 1.0–1.8, *p* = 0.09). After removal of cerebrovascular risk factors, the RR of ischemic stroke remained increased in COVID-19 inpatients (RR = 3.4; 95%CI: 1.4–8.2 after 1 month; RR = 3.0; 95%CI: 1.5–6.3 after 3 months; RR = 3.5; 95%CI: 1.7–7.2 after 6 months; RR = 2.8; 95%CI: 1.5–5.0 after 12 months; Supplementary Table 6).

The frequency of ischemic stroke did not differ significantly between COVID-19 positive and bacterial pneumonia inpatients (Table 3). After removal of individuals with significant cerebrovascular risk factors, there remained no significant difference between groups (Supplementary Table 6). After stratification for age, the incidence of ischemic stroke was increased in COVID-19 positive inpatients aged ≥ 80 (RR = 2.7; 95%CI: =1.2–6.2), but not in other age groups.

### Intracerebral and Subarachnoid Hemorrhage

The RR of intracerebral hemorrhage was increased 12 months after a positive test in COVID-19 positive compared to COVID-negative outpatients (RR = 4.8; 95%CI: 1.8–12.9). There were no other differences in the rates of intracerebral and subarachnoid hemorrhage between groups (Tables 2, 3 and Supplementary Tables 4, 5). Notably, COVID-19 outpatients received higher rates of intravenous thrombolysis, a risk factor for medically induced intracerebral hemorrhage (21) (0.14% in COVID-19 positive vs. 0.02% in COVID-negative outpatients, χ^2^ = 177.6, *p* < 0.0001). After exclusion of those treated with intravenous thrombolysis, the RR of intracerebral hemorrhage remained elevated after 12 months (RR = 4.4, 95%CI 1.6–11.5). There were too few cases to carry out meaningful statistics after 1–6 months.

### Multiple Sclerosis and Other Auto-Immune Disorders

The frequency of new-onset multiple sclerosis did not differ significantly between COVID-19 positive and COVID-negative individuals (in- and outpatients combined), nor separately across in- and outpatients (Table 2 and Supplementary Table 3). There was also no significant difference in multiple sclerosis rates between COVID-19 positive inpatients and influenza inpatients (Table 3), and there were not enough cases to conduct meaningful statistics in pneumonia inpatients.

Among 43,375 COVID-19 individuals, one developed Guillain-Barré syndrome within 1 month (0.002%) and two (0.005%) within 3 months. One individual (0.002%) developed myasthenia gravis one through 12 months, and none (0.0%) developed narcolepsy (Tables 2, 3). There were not enough cases to conduct meaningful comparisons between groups.

## Discussion

Key findings from this population-based cohort study covering roughly half of Denmark's population include an increased frequency of new-onset neurodegenerative and cerebrovascular (but not neuroimmune) disorders in COVID-19 positive compared to COVID-negative individuals. However, when comparing the frequencies of these disorders after COVID-19 with those after influenza and community-acquired pneumonia, we found no significant differences, except for ischemic stroke.

### Neurodegenerative Diseases

Alzheimer's disease was 3.4 times more frequent and Parkinson's disease was 2.2 times more frequent in COVID-19 positive than COVID-negative individuals, 12 months after a COVID-19 test. These findings should be considered in light of the prolonged temporal course and the complex pathophysiology of these disorders, including a possible role for neuroinflammation: it is hypothesized that the body's innate response and subsequent inflammatory processes can induce a toxic cycle of accumulating β-amyloid and alpha-synuclein peptides (the pathologic hallmarks of Alzheimer's and Parkinson's diseases) (22–26). In support of this, unexpectedly high amounts of β-amyloid peptides have been discovered in brain autopsies of young deceased patients with COVID-19 (27). Other factors such as fatigue, depression, and anxiety after COVID-19 may also contribute to the development of neurodegenerative disorders (20, 28–34). Moreover, it is uncertain if the risk of Alzheimer's disease and Parkinson's disease differs after COVID-19 compared to after influenza and bacterial pneumonia. Finally, the scientific focus on long-term sequelae after COVID-19 may have led to increased recognition by clinicians and hence earlier diagnosis, perhaps explaining some of the observed increase in neurodegenerative diagnoses.

### Cerebrovascular Disorders

#### Ischemic Stroke

New-onset ischemic stroke was 2.3 times more frequent in COVID-19 positive than COVID-negative outpatients after 3 months. Ischemic stroke was also 1.7 times more frequent in COVID-19 inpatients compared to influenza inpatients in the early and subacute phases after a positive test, as supported by previous retrospective studies (albeit with shorter observation periods) (5, 35). Ischemic stroke was also 2.7 times more frequent in COVID-19 inpatients compared to bacterial pneumonia among the elderly. In our study, the overall incidence of ischemic stroke in COVID-19 positive inpatients (1.8%) is well in line with previously reported data (0.4-2.7%) (36–39). Of note, age-specific stratifications showed that the relative risk for ischemic stroke was highest amongst patients between 40 and 59 years. A recent study of 37,379 Medicare fee-for-service beneficiaries aged ≥65 years diagnosed with COVID-19 (36) and a multi-center study involving a further 423 patients (40) similarly found an increase in ischemic stroke among younger patients when compared to population studies before the pandemic.

Increased rates of ischemic stroke in COVID-19 patients may occur for several reasons. In line with an inflammatory etiology, there were minimal differences in cerebrovascular events between COVID-19 positive and community-acquired pneumonia inpatients in our study, except for elderly patients, who generally have a weaker inflammatory response (41). It is unknown if the increased risk of thromboembolic events in COVID-19 patients can be directly attributed to unique properties of the virus, or if it is a consequence of a more pronounced inflammatory state (41). Moreover, given the association of COVID-19 with cardiac disorders, including myocarditis, arrhythmias, heart failure, and myocardial infarction, cardiac embolism is also a potential mechanism (42–44). It should be noted that COVID-19 patients had a slightly higher rate of certain pre-existing risk factors for ischemic stroke, including hypercholesterolemia, diabetes mellitus, and hypertension, as have previously been reported (3, 45). However, even when these cerebrovascular risk factors were excluded from analysis, the COVID-19 population maintained a higher risk of ischemic stroke. Finally, factors such as immobilization during hospital admission may increase stroke risk as well (44).

#### Intracerebral and Subarachnoid Hemorrhage

The 1-month incidence of intracerebral hemorrhage among COVID-19 inpatients was 0.1%, similar to previously published studies (46). The frequency was 4.8 times higher in COVID-19 positive compared to negative outpatients. There was, however, no excess risk compared to patients with influenza or community-acquired bacterial pneumonia. Some authors have argued that a subset of intracerebral hemorrhages may be due to hemorrhagic conversion of ischemic events, particularly after anticoagulation therapy (47–49). In two recent studies, 76% (25 of 33) and 60% (6 out 10) of patients developed intracerebral hemorrhage after low- or high-dose anticoagulation therapy (47, 48). Besides anticoagulation, a systematic review of 94 studies found that older age, mechanical ventilation and extracorporeal membrane oxygenation also increased the risk of intracranial hemorrhage in COVID-19 patients (46). In our study, the risk of intracerebral hemorrhage remained elevated after removal of patients who received intravenous thrombolysis, indicating an independent COVID-19 related risk.

In our study of over 43,000 COVID-19 patients, only four individuals developed subarachnoid hemorrhage within the first month, and 10 within 12 months. This does not represent an excess risk compared to COVID-negative individuals and patients with influenza or bacterial pneumonia. Our results confirm findings from another large study with 85,645 COVID-19 patients, in which 86 developed SAH, without an excess risk compared to COVID-negative patients (50).

### Auto-Immune Neurological Diseases

#### Guillain-Barré Syndrome

In our study, only two patients developed GBS. In a study of 1,200 COVID-19 patients from Italy (51) and a study of 3,927 COVID-19 patients from India, there were five cases of GBS each, (52) which appears to be an order of magnitude higher than our data. Another epidemiologic study showed that the incidence of GBS was lower during the pandemic than the corresponding months in the four preceding pre-pandemic years (53). However, precautionary measures intended to reduce the risk of COVID-19 transmission might also have reduced the rate of other infectious diseases associated with GBS (54).

#### Multiple Sclerosis

In the COVID-19 positive population, 14 of 43,375 individuals developed MS 12 months after a positive test, which did not represent an excess risk. Cases of multiple sclerosis after COVID-19 infection or vaccination have been reported, (55–59) but to our knowledge, no study has yet investigated the incidence of multiple sclerosis after COVID-19.

#### Myasthenia Gravis and Narcolepsy

In the COVID-19 cohort, only one individual developed myasthenia gravis, and none were diagnosed with narcolepsy, 12 months after a positive test. Only a few cases of new-onset myasthenia gravis following COVID-19 have been reported, (60, 61) and to our knowledge, none of narcolepsy. Based on our findings, it appears that COVID-19 does not increase the 1-year risk of myasthenia gravis or narcolepsy. It must, however, be kept in mind that the median age of new-diagnosed narcolepsy patients is 12 years (62). Given the inclusion criteria of adults ≥18 years, we may have missed a possible association between COVID-19 and narcolepsy. Longer follow-up studies in larger and younger COVID-19 populations are needed to exclude subsequent risks of myasthenia gravis and narcolepsy.

### Strengths and Limitations

The strengths of this study include the large population and wide catchment area, constituting half of the Danish population. We were able to include all individuals irrespective of age, sex, ethnicity, lifestyle, and socioeconomic background without loss-to-follow-up. Sensitivity analyses showed no differences in rates of clinical work-ups utilizing cerebral PET-FDG-18 and SPECT for diagnoses of neurodegenerative disorders, nor in the rates of risk factors for auto-immune disorders.

Given the nature of aggregated data, several caveats need to be considered. First, we could not adjust for potential confounders such as socioeconomic, lifestyle, pre-existing comorbidities, and length of hospitalization. Instead, we stratified analyses by age, sex, smoking status and pre-existing comorbidities.

Second, we only captured a subset of the Danish population's absolute number of tested individuals, because only COVID-19 tests performed in hospital facilities are registered in the Danish electronic health record system, and not those performed in the community setting (including over-the-counter antigen tests or PCR tests from private providers and the primary care sector). Altogether, we captured ~20% of the COVID-19 positive (17) and 40% of the COVID-negative (18) population in the Capital Region and Region Zealand (which together correspond to roughly half the population in Denmark).

To assess the representativeness of our study population, we compared the frequencies of neurological diseases in our COVID-negative population with those of the general Danish population. We found that the prevalence or incidences of Alzheimer's disease, Parkinson's disease, narcolepsy, and intracerebral hemorrhage were representative of the Danish and other Western populations (Supplementary Table 7) (63). However, the prevalence of ischemic stroke, subarachnoid hemorrhage, and GBS were higher than previous reports from Denmark (64–66). While these results may be surprising, they are in line with a recent Danish study of 23,688 individuals that showed an increase in ischemic stroke in the pandemic period from March 13, 2020 – February 28, 2021, (67) and another showing increasing rates of GBS from 2019 to 2020 (68). The incidence of multiple sclerosis was also higher than the reported yearly incidence in the Danish population, (69) and may be accounted for by the younger population in the Greater Copenhagen area (18) and, possibly, by greater air pollution in urban areas (70–72). Altogether, however, these figures suggest that our study cohorts are representative of the general Danish population.

Given the attention on COVID-19 in the medical community, the frequency of neurological diagnoses may have been increased during the pandemic, thereby artificially increasing the numbers in our study. Conversely, we may have missed the diagnosis of some neurologic cases given the nature of aggregated data from electronic health records and the one-year follow-up duration which arguably is too short to detect longer-term changes, as might be the case for multiple sclerosis after Ebstein-Barr virus infection (73).

## Conclusion

In this population-based study covering ~50% of the Danish population, we found support for an increased risk of neurodegenerative disorders (i.e., Alzheimer's disease and Parkinson's disease) and cerebrovascular disorders (i.e., ischemic stroke and intracerebral hemorrhage), in COVID-19 patients compared to individuals tested negative for COVID-19. While the risk of ischemic stroke was increased with COVID-19 compared to influenza, reassuringly, most neurological disorders do not appear to be more frequent after COVID-19 than after influenza or community-acquired bacterial pneumonia. Future nationwide registry-based studies of pre-and post-pandemic disease rates with full nationwide follow-up are required to confirm these observations.

## Data Availability Statement

The original contributions presented in the study are included in the article/Supplementary Material, further inquiries can be directed to the corresponding authors.

## Author Contributions

PZ, CP, MB, and DK contributed to the conception and design of the study. PZ and CP extracted data, performed statistical analyses, and drafted the first version of the manuscript. All authors contributed to manuscript revision and approved the submitted version.

## Funding

This research was supported by grants from Novo Nordisk (Grant Number NNF21OC0067769) and the Lundbeck Foundation (Grant Number R349-2020-658).

## Conflict of Interest

The authors declare that the research was conducted in the absence of any commercial or financial relationships that could be construed as a potential conflict of interest.

## Publisher's Note

All claims expressed in this article are solely those of the authors and do not necessarily represent those of their affiliated organizations, or those of the publisher, the editors and the reviewers. Any product that may be evaluated in this article, or claim that may be made by its manufacturer, is not guaranteed or endorsed by the publisher.
