# Supplementary material for: Frequency of Neurological Diseases After COVID-19, Influenza A/B and Bacterial Pneumonia
Source: Front Neurol. 2022 Jun 23;13:904796. doi: 10.3389/fneur.2022.904796 (PMC9259944; doi:10.3389/fneur.2022.904796)
Supplement: Supplementary file 1 [file Table_1.docx]

**Supplementary Tables and Figures**

1. Table S1- Search strings and ICD-10 diagnostic codes

2. Table S2 – Comparison of risk factors across groups

3. Table S3 - Age and sex stratification

4. Table S4 – Outpatients - inpatients stratification, COVID-19

5. Table S5 –Outpatients-inpatients combined, Influenza A/B

6. Table S6 – Stratification after removal of risk factors

7. Table S7 - Comparison of the incidence or prevalence of neurologic disorders in the COVID-19 negative population to the background population

8. Figure 1 – Flowchart of individuals tested for COVID-19 or Influenza A/B, and diagnosed with community-acquired bacterial pneumonia

**1. Supplementary table 1 (S1)**

Search strategies used in the Slicer Dicer function of EPIC and list of ICD-10 codes used to identify comorbidities, new-onset neurological disorders and diagnostic processes.

| GROUPS | TIME PERIOD | SEARCH STRINGS |
| --- | --- | --- |
| COVID-19 positive | 27 February 2020- 27 November 2021 | All patients **≥** 18 years **AND** (laboratory findings of *positive* SARS-COV-2 RNA **OR** coronavirus (2019-ncov) **OR** coronavirus 2019-ncov **OR** SARS-CoV-2 (POC) **OR** SARS-CoV-2 (GENEXPERT) **OR** SARS-CoV-2 SSI100163) |
| COVID-19 negative |  | All patients **≥** 18 years **AND** (laboratory findings of *negative* SARS-COV-2 RNA  **OR** coronavirus (2019-ncov) **OR** coronavirus 2019-ncov **OR** SARS-CoV-2 (POC) **OR** SARS-CoV-2 (GENEXPERT) **OR** SARS-CoV-2 SSI100163) **AND** (exclusion of COVID-19 positive group) |
| Community-acquired pneumonia | 27 February 2020- 27 November 2021 | All patients **≥** 18 years **AND** (diagnosis of bacterial pneumonia) **AND** (exclusion of COVID-19 positive group) **AND** (exclusion of bacterial pneumonia preceded by COVID-19 positive up to 1 year) |
| Influenza A/B | 27 February 2018- 27 November 2020 | All patients ≥ 18 years **AND** (laboratory findings of *positive* Influenza A. Influenza B. Influenza A RNA. Influenza B RNA. Influenza VIRUS B. Influenza TYPE A. Influenza TYPE B. Influenza A RNA (POC) OR Influenza B RNA (POC)) |
| DEMOGRAPHICS | | |
| *Sex* | |  |
| Female/Male | | Use of slicing function for male or female in all GROUPS |
| *Age* | | |
| Mean | | Use of measure function to obtain mean in all GROUPS |
| Range | | Use of slicing function for age in all GROUPS |
| *Admission status* | | |
| Inpatient | | GROUP as described **PLUS** overlapping criteria of hospitalization at the same time as diagnosis or laboratory test |
| Outpatient | | GROUP as described **AND** (exclusion of inpatient as above) |
| COMORBIDITIES | | |
| History of previous comorbidities | | GROUP as described **PLUS** sequential criteria of present diagnoses up to 100 years prior to pneumonia diagnosis or COVID-19 or Influenza laboratory test |
| Smoking | | GROUP as described **AND (**current smoker OR previous smoker) |
| New onset neurological diseases | | GROUP as described **AND** (exclusion of present diagnosis between 1/1/1980 and 26/2/2020) **PLUS** sequential criteria of diagnosis 1.3.6. and 12 months after bacterial pneumonia diagnosis or COVID-19 or Influenza laboratory test |
| List of ICD-10 codes | | |
| Disease | | |
| Alzheimer’s disease | | DG309 |
| Celiac disease | | DK900 |
| Delirium | | DF059 |
| Dementia | | DF039 |
| Diabetes mellitus type 1 | | DE10 + DE109A |
| Diabetes mellitus type 2 | | DE11 + DE119A |
| Hashimoto’s auto-immune thyroiditis | | DE063A |
| Hypercholesterolemia | | DE780 |
| Hypertension | | DI109 |
| Ischemic stroke | | DI639 |
| Intracerebral hemorrhage | | DI612 |
| Guillian-Barré syndrome | | DG610 |
| Multiple sclerosis | | DG359 |
| Myasthenia gravis | | DG700 |
| Narcolepsy | | DG474 |
| Parkinson’s disease | | DG209 |
| Rheumatoid arthritis | | DM069 |
| Subarachnoid hemorrhage | | DI609 |
| Transitory cerebral ischemia | | DG459 |
| Diagnostic processes | | |
| PET-CT (18F-FDG) | | BLD594 + BLD621 |
| SPECT | | BLD506 + PRO270 |

**2. Supplementary table 2 (S2)**

Comparison of risk factors among all groups. Chi-square with Yates correction was used to this purpose.

|  | Inpatients + outpatients | | Inpatients | | | Outpatients |
| --- | --- | --- | --- | --- | --- | --- |
|  | COVID-19 positive  vs negative  (𝜒2; p value) | COVID-19 positive vs influenza  (𝜒2; p value) | COVID-19 positive vs negative  (𝜒2; p value) | COVID-19 positive vs influenza  (𝜒2; p value) | COVID-19 positive vs pneumonia  (𝜒2; p value) | COVID-19 positive vs negative  (𝜒2; p value) |
| Hashimoto’s thyroiditis | 0.06; 0.8 | 2.2; 0.1 | 0.6; 0.4 | <0.00001; 1 | N/A | 0.5; 0.4 |
| Celiac disorder | 1.04; 0.3 | 3.9; 0.05 | 0.1; 0.7 | 2.5; 0.1 | 0.04; 0.8 | 0.8; 0.4 |
| Type 1 DM | 1.5; 0.2 | 2.0; 0.2 | 0.8; 0.4 | <0.00001; 1 | 0.09; 0.76 | 2.6; 0.1 |
| Rheumatoid arthritis | 1.8; 0.2 | 4.3; 0.04* | 3.6; 0.06 | 0.04; 0.8 | 1.5; 0.2 | 2.1; 0.1 |
| Transitory ischemic attack | 0.9; 0.3 | 15.1; 0.0001* | 1.6; 0.2 | 21.2; <0.00001* | 5.5; 0.02* | 45.5; <0.00001* |
| Hypercholestero  -lemia | 12.2; 0.0005* | 3.1; 0.08 | 28.9; <0.0001* | 40.1; <0.00001* | 0.1; 0.75 | 22.9; <0.00001* |
| Type 2 DM | 168.3; < 0.00001* | 0.6; 0.4 | 1.9;6.5 | 43.8; <.0.00001* | 0.98; 0.3 | 126.8; <0.00001* |
| Obesity | 0.03; 0.8 | 61.4; <0.00001* | 1.6; 0.2 | 71.8; 0.00001* | 12.1; 0.0005* | 14.2; 0.0002* |
| Hypertension | 88.7; < 0.00001* | 1.5; 0.2 | 145 ; < 0.0001* | 132.5; <0.00001* | 35.2; <0.0001* | 158.4; <0.00001* |
| Smoking | 148; < 0.00001* | 902.3; <0.00001* | 4.9; 0.03* | 114.2; <0.00001* | 153.4; p<0.0001* | 11.7; 0.0006* |
| Delirium | 128.2; <0.00001* | 16.8; <0.00001* | 53.1; <0.0001* | 37.7; <0.00001* | 1.0; 0.3 | 348.5;<0.00001* |

* Statistically significant 𝜒2 (p<0.05)

DM = diabetes mellitus

**3. Supplementary table 3 (S3)**

Relative risk of of neurodegenerative, cerebrovascular, and immune-mediated disorders stratified for age and sex across all groups, twelve months after a test for influenza, COVID-19 or a diagnosis of community acquired pneumonia.

| Alzheimer’s disease | | | | | | | | | | | | | | | | | | | |
| --- | --- | --- | --- | --- | --- | --- | --- | --- | --- | --- | --- | --- | --- | --- | --- | --- | --- | --- | --- |
| Age / Sex | Covid + | Influenza | RR (95%CI) | Covid + inpt | Influenza  inpt | RR  (95%CI) | Covid + inpt | Pneumo  nia | RR  (95% C.I.) | Covid + inpt | Covid - inpt | RR (95% C.I.) | Covid + outpt | Covid - outpt | RR (95% C.I.) | Covid + | Covid - | RR (95% C.I.) |  |
| 18-39 | 0 | 0 | N/A | 0 | 0 | N/A | 0 | 0 | N/A | 0 | 0 | N/A | 0 | 0 | N/A | 0 | 0 | N/A |  |
| 40-59 | 0 | 0 | N/A | 0 | 0 | N/A | 0 | 0 | N/A | 0 | 0 | N/A | 0 | 1 | N/A | 0 | 1 | N/A |  |
| 60-79 | 15 | 3 | N/A | 5 | 3 | N/A | 5 | 0 | N/A | 5 | 104 | 1.2  (0.5-2.9) | 10 | 49 | 4.6  (2.3-9.0)* | 15 | 153 | 2.3  (1.3-3.9)* |  |
| >80 | 24 | 11 | 0.6  (0.3-1.1) | 12 | 9 | 0.7  (0.3-1.6) | 11 | 1 | N/A | 12 | 178 | 1.3  (0.8-2.4) | 12 | 66 | 1.6  (0.9-3.0) | 24 | 244 | 1.3  (0.9-2.0) |  |
| Males | 16 | 6 | 0.5  (0.2-1.3) | 10 | 4 | 1.1  (0.4-3.4) | 10 | 1 | N/A | 10 | 99 | 2.3  (1.2-4.3)* | 6 | 45 | 2.6  (1.1-6.0)* | 16 | 144 | 2.2  (1.3-3.7)* |  |
| Females | 23 | 8 | 0.5  (0.2-1.2) | 7 | 8 | 0.6  (0.2-1.5) | 6 | 0 | N/A | 7 | 183 | 1.4(0.7-3.0) | 16 | 71 | 4  (2.3-6.8)* | 23 | 254 | 1.8  (1.2-2.8)* |  |
| Parkinson’s disease | | | | | | | | | | | | | | | | | | | |
| 18-39 | 0 | 0 | N/A | 0 | 0 | N/A | 0 | 0 | N/A | 0 | 0 | N/A | 0 | 0 | N/A | 0 | 0 | N/A |  |
| 40-59 | 3 | 0 | N/A | 0 | 0 | N/A | 0 | 1 | N/A | 0 | 14 | N/A | 3 | 22 | N/A | 3 | 36 | N/A |  |
| 60-79 | 11 | 9 | 0.4  (0.2-0.9) | 4 | 7 | 0.3  (0.1-1.1) | 5 | 4 | 0.3  (0.1-1.0) | 5 | 204 | 0.6  (0.2-1.5) | 8 | 104 | 1.7  (0.8-3.5) | 13 | 306 | 1.0  (0.6-1.7) |  |
| >80 | 17 | 4 | 1.1  (0.4-3.2) | 5 | 4 | 0.6  (0.2-2.4) | 5 | 3 | N/A | 5 | 161 | 0.6  (0.3-1.5) | 12 | 37 | 2.9  (1.5-5.4)* | 17 | 198 | 1.2  (0.7-1.9) |  |
| Males | 16 | 10 | 0.3  (0.1-0.6) | 4 | 8 | 0.2  (0.1-0.7) | 5 | 7 | 0.1  (0.04-0.4) | 5 | 138 | 0.8  (0.3-2.0) | 13 | 101 | 2.5  (1.4-4.0)* | 18 | 323 | 1.1  (0.7-1.8) |  |
| Females | 15 | 3 | N/A | 5 | 3 | N/A | 5 | 1 | N/A | 5 | 110 | 1.7  (0.7-4.1) | 10 | 62 | 2.8  (1.4-5.5) | 15 | 217 | 1.4  (0.8-1.4) |  |
| Ischemic stroke | | | | | | | | | | | | | | | | | | | |
| 18-39 | 2 | 0 | N/A | 2 | 0 | N/A | 2 | 1 | N/A | 2 | 115 | N/A | 0 | 22 | N/A | 1 | 137 | N/A |  |
| 40-59 | 32 | 4 | 1.3  (0.5-3.7) | 9 | 2 | N/A | 9 | 4 | 0.1  (0.1-0.6) | 9 | 1078 | 0.2  (0.1-0.4) | 23 | 179 | 2.4  (1.6-3.7)* | 32 | 1284 | 0.5  (0.3-0.7) |  |
| 60-79 | 137 | 34 | 1.2  (0.8-1.8) | 78 | 31 | 1.4  (0.9-2.1) | 77 | 17 | 1.0  (0.6-1.7) | 79 | 3726 | 0.5  (0.4-0.6) | 59 | 515 | 2.6  (2.0-3.4)* | 139 | 4214 | 0.7  (0.6-0.9) |  |
| >80 | 108 | 27 | 1.0  (0.7-1.6) | 56 | 25 | 1.2  (0.7-1.8) | 51 | 6 | 2.7  (1.2-6.2) * | 56 | 2083 | 0.5  (0.4-0.7) | 53 | 194 | 2.4  (1.8-3.2)* | 109 | 2275 | 0.6  (0.5-0.8) |  |
| Males | 153 | 32 | 0.9  (0.6-1.3) | 82 | 29 | 1.2  (0.8-1.8) | 78 | 19 | 0.8  (0.5-1.3) | 82 | 3818 | 0.5  (0.4-0.6) | 72 | 499 | 2.8  (2.2-3.5)* | 154 | 4316 | 0.7  (0.6-0.8) |  |
| Females | 126 | 33 | 0.7  (0.5-1.1) | 63 | 29 | 1.4  (0.9-2.1) | 61 | 9 | 1.2  (0.6-2.4) | 64 | 3184 | 0.7  (0.6-0.9) | 63 | 411 | 2.7  (2.1-3.5)* | 127 | 3594 | 0.7  (0.6-0.9) |  |
| Intracerebral hemorrhage | | | | | | | | | | | | | | | | | | | |
| 18-39 | 0 | 0 | N/A | 0 | 0 | N/A | 0 | 0 | N/A | 0 | 8 | N/A | 0 | 6 | N/A | 0 | 14 | N/A |  |
| 40-59 | 4 | 0 | N/A | 2 | 0 | N/A | 2 | 0 | N/A | 2 | 61 | N/A | 2 | 3 | N/A | 4 | 64 | 1.2  (0.4-3.4) |  |
| 60-79 | 6 | 0 | N/A | 4 | 0 | N/A | 4 | 0 | N/A | 4 | 158 | 0.6  (0.2-1.7) | 2 | 9 | N/A | 6 | 167 | 0.8  (0.4-1.9) |  |
| >80 | 6 | 1 | N/A | 5 | 1 | N/A | 5 | 0 | N/A | 5 | 91 | 1.1  (0.4-2.7) | 1 | 4 | N/A | 6 | 95 | 0.9  (0.4-1.9) |  |
| Males | 8 | 0 | N/A | 5 | 0 | N/A | 5 | 0 | N/A | 5 | 169 | 0.7  (0.3-1.6) | 3 | 11 | N/A | 8 | 180 | 0.9  (0.4-1.8) |  |
| Females | 8 | 1 | N/A | 6 | 1 | N/A | 6 | 0 | N/A | 6 | 149 | 1.5  (0.7-3.4) | 2 | 11 | N/A | 8 | 160 | 1.0  (0.5-2.1) |  |
| Subarachnoid hemorrhage | | | | | | | | | | | | | | | | | | | |
| 18-39 | 0 | 0 | N/A | 0 | 0 | N/A | 0 | 0 | N/A | 0 | 20 | N/A | 0 | 6 | N/A | 0 | 23 | N/A |  |
| 40-59 | 4 | 1 | N/A | 3 | 0 | N/A | 3 | 0 | N/A | 3 | 80 | N/A | 1 | 12 | N/A | 4 | 92 | 0.9  (0.3-2.3) |  |
| 60-79 | 4 | 1 | N/A | 2 | 0 | N/A | 2 | 0 | N/A | 2 | 106 | N/A | 2 | 8 | N/A | 4 | 114 | 0.8  (0.3-2.2) |  |
| >80 | 2 | 0 | N/A | 2 | 0 | N/A | 2 | 0 | N/A | 2 | 58 | N/A | 0 | 4 | N/A | 2 | 62 | N/A |  |
| Males | 9 | 1 | N/A | 6 | 0 | N/A | 6 | 0 | N/A | 6 | 113 | 1.2  (0.5-2.7) | 3 | 15 | N/A | 9 | 128 | 1.4  (0.7-2.8) |  |
| Females | 1 | 1 | N/A | 1 | 0 | N/A | 1 | 0 | N/A | 1 | 148 | N/A | 0 | 15 | N/A | 1 | 163 | N/A |  |
| Guillain-Barré syndrome | | | | | | | | | | | | | | | | | | | |
| 18-39 | 0 | 0 | N/A | 0 | 0 | N/A | 0 | 0 | N/A | 0 | 13 | N/A | 0 | 2 | N/A | 0 | 14 | N/A |  |
| 40-59 | 0 | 2 | N/A | 0 | 2 | N/A | 0 | 0 | N/A | 0 | 15 | N/A | 0 | 3 | N/A | 0 | 19 | N/A |  |
| 60-79 | 2 | 0 | N/A | 1 | 0 | N/A | 1 | 1 | N/A | 1 | 26 | N/A | 1 | 2 | N/A | 2 | 28 | N/A |  |
| >80 | 0 | 0 | N/A | 0 | 0 | N/A | 0 | 0 | N/A | 0 | 3 | N/A | 0 | 0 | N/A | 0 | 3 | N/A |  |
| Males | 1 | 2 | N/A | 0 | 0 | N/A | 0 | 1 | N/A | 0 | 29 | N/A | 1 | 3 | N/A | 1 | 32 | N/A |  |
| Females | 1 | 0 | N/A | 1 | 0 | N/A | 1 | 0 | N/A | 1 | 28 | N/A | 0 | 4 | N/A | 1 | 32 | N/A |  |
| Multiple sclerosis | | | | | | | | | | | | | | | | | | | |
| 18-39 | 0 | 1 | N/A | 0 | 0 | N/A | 0 | 0 | N/A | 0 | 118 | N/A | 5 | 69 | 1.3  (0.5-3.2) | 5 | 162 | 0.66 |  |
| 40-59 | 2 | 0 | N/A | 0 | 0 | N/A | 0 | 0 | N/A | 0 | 52 | N/A | 3 | 63 | N/A | 3 | 117 | N/A |  |
| 60-79 | 2 | 2 | N/A | 1 | 2 | N/A | 1 | 2 | N/A | 1 | 29 | N/A | 4 | 17 | 5.2 (1.8-15.7)* | 5 | 45 | 2.6  (1.0-6.5) |  |
| >80 | 0 | 0 | N/A | 0 | 0 | N/A | 0 | 0 | N/A | 0 | 8 | N/A | 1 | 0 | N/A | 1 | 8 | N/A |  |
| Males | 1 | 2 | N/A | 0 | 1 | N/A | 0 | 2 | N/A | 0 | 69 | N/A | 3 | 38 | N/A | 3 | 107 | N/A |  |
| Females | 3 | 1 | N/A | 1 | 1 | N/A | 1 | 0 | N/A | 1 | 115 | N/A | 10 | 111 | 1.6  (0.8-3) | 11 | 225 | 1.0  (0.5-1.8) |  |
| Myasthenia gravis | | | | | | | | | | | | | | | | | | | |
| 18-39 | 0 | 0 | N/A | 0 | 0 | N/A | 0 | 0 | N/A | 0 | 4 | 0 | 0 | 10 | N/A | 0 | 14 | N/A |  |
| 40-59 | 0 | 0 | N/A | 0 | 0 | N/A | 0 | 0 | N/A | 0 | 5 | 0 | 0 | 5 | N/A | 0 | 10 | N/A |  |
| 60-79 | 0 | 0 | N/A | 0 | 0 | N/A | 0 | 0 | N/A | 0 | 22 | 0 | 0 | 12 | N/A | 0 | 34 | N/A |  |
| >80 | 1 | 0 | N/A | 0 | 0 | N/A | 1 | 0 | N/A | 1 | 10 | N/A | 0 | 3 | N/A | 1 | 13 | N/A |  |
| Males | 1 | 0 | N/A | 1 | 0 | N/A | 1 | 0 | N/A | 1 | 22 | N/A | 0 | 10 | N/A | 1 | 32 | N/A |  |
| Females | 0 | 0 | N/A | 0 | 0 | N/A | 0 | 0 | N/A | 0 | 19 | N/A | 0 | 20 | N/A | 0 | 39 | N/A |  |
| Narcolepsy | | | | | | | | | | | | | | | | | | | |
| 18-39 | 0 | 0 | N/A | 0 | 0 | N/A | 0 | 0 | N/A | 0 | 6 | N/A | 0 | 18 | N/A | 0 | 24 | N/A |  |
| 40-59 | 0 | 0 | N/A | 0 | 0 | N/A | 0 | 0 | N/A | 0 | 3 | N/A | 0 | 9 | N/A | 0 | 12 | N/A |  |
| 60-79 | 0 | 0 | N/A | 0 | 0 | N/A | 0 | 0 | N/A | 0 | 3 | N/A | 0 | 0 | N/A | 0 | 3 | N/A |  |
| >80 | 0 | 0 | N/A | 0 | 0 | N/A | 0 | 0 | N/A | 0 | 1 | N/A | 0 | 1 | N/A | 0 | 2 | N/A |  |
| Males | 0 | 0 | N/A | 0 | 0 | N/A | 0 | 0 | N/A | 0 | 5 | N/A | 0 | 8 | N/A | 0 | 13 | N/A |  |
| Females | 0 | 0 | N/A | 0 | 0 | N/A | 0 | 0 | N/A | 0 | 8 | N/A | 0 | 20 | N/A | 0 | 28 | N/A |  |

Statistical analyses were only conducted for diseases with ≥ 4 cases in each group.

* Statistically significant RR (p<0.05)

**4. Supplementary table 4 (S4)**

Relative risk of neurologic immune-mediated,neurodegenerative, and cerebrovascular disorders in COVID-19positive compared to COVID-19 negative individuals stratified for inpatients and outpatients.

|  | Inpatients | | | Outpatients | | |
| --- | --- | --- | --- | --- | --- | --- |
|  | COVID-19  positive  (n=8,013) | COVID-19  negative  (n=230,686) | RR (95%CI) | COVID-19  positive  (n=35,362) | COVID-19  negative  (n=645,670) | RR (95%CI) |
| 1 month (n,%) | | | | | | |
| Alzheimer’s disease^♦^ | **-** | **-** | **-** | 7 (0.02%) | 42 (0.007%) | 3.0 (1.4-6.8) * |
| Parkinson’s disease^♦^ | - | - | - | 6 (0.02%) | 69 (0.01%) | 1.6 (0.3-7.6) |
| Ischemic stroke | 85 (1.06%) | 5,825 (2.53%) | 0.4 (0.3-0.5) | 32 (0.09%) | 426 (0.07%) | 1.4 (1.0-2.0) |
| Intracerebral hemorrhage | 6 (0.08%) | 243 (0.11%) | 0.7 (0.3-1.6) | 1 (0.003%) | 7 (0.001%) | N/A |
| Subarachnoid  hemorrhage | 4 (0.05%) | 185 (0.08%) | 0.6 (0.2-1.7) | 0 (0.00%) | 16 (0.002%) | N/A |
| Guillain-Barré syndrome | 1 (0.01%) | 49 (0.02%) | N/A | 0 (0.00%) | 3 (0.00%) | N/A |
| Multiple sclerosis | 1 (0.01%) | 122 (0.05%) | N/A | 3 (0.008%) | 63 (0.01%) | N/A |
| Myasthenia gravis | 1 (0.01%) | 28 (0.01%) | N/A | 0 (0.00%) | 16 (0.002%) | N/A |
| Narcolepsy | 0 (0.00%) | 4 (0.002%) | N/A | 0 (0.00%) | 14 (0.002%) | N/A |
| 3 months (n,%) | | | | | | |
| Alzheimer’s disease^♦^ | - | - | - | 12 (0.03%) | 66 (0.01%) | 3.3 (1.8-6.1) * |
| Parkinson’s disease^♦^ | - | - | - | 14 (0.04%) | 109 (0.02%) | 2.3 (1.3-4.1) * |
| Ischemic stroke | 113 (1.41%) | 6,372 (2.76%) | 0.5 (0.4-0.6) | 67 (0.19%) | 536 (0.08%) | 2.3 (1.8-3.0) * |
| Intracerebral hemorrhage | 8 (0.1%) | 273 (0.12%) | 0.8 (0.4-1.7) | 2 (0.006%) | 9 (0.001%) | N/A |
| Subarachnoid  hemorrhage | 5 (0.06%) | 214 (0.09%) | 0.7 (0.3-1.6) | 0 (0.00%) | 19 (0.003%) | N/A |
| Guillain-Barré syndrome | 1 (0.01%) | 54 (0.02%) | N/A | 0 (0.00%) | 4 (0.001%) | N/A |
| Multiple sclerosis | 1 (0.01%) | 153 (0.07%) | N/A | 5 (0.01%) | 93 (0.01%) | 1.0 (0.4-2.4) |
| Myasthenia gravis | 1 (0.01%) | 36 (0.02%) | N/A | 0 (0.00%) | 23 (0.004%) | N/A |
| Narcolepsy | 0 (0.00%) | 10 (0.004%) | N/A | 0 (0.00%) | 20 (0.003%) | N/A |
| 6 months | | | | | | |
| Alzheimer’s disease | 4 (0.05%) | 35 (0.02%) | 3.3 (1.2-9.3) * | 17 (0.05%) | 86 (0.01%) | 3.6 (2.1-6.1) * |
| Parkinson’s disease | 1 (0.01%) | 41 (0.02%) | N/A | 19 (0.05%) | 129 (0.02%) | 2.7 (1.7-4.4) * |
| Ischemic stroke | 128 (1.60%) | 6,710 (2.90%) | 0.5 (0.5-0.7) | 99 (0.28%) | 656 (0.10%) | 2.8 (2.2-3.4) * |
| Intracerebral hemorrhage | 10 (0.13%) | 295 (0.13%) | 1.0 (0.5-1.8) | 3 (0.008%) | 11 (0.002%) | N/A |
| Subarachnoid  hemorrhage | 5 (0.06%) | 233 (0.10%) | 0.6 (0.3-1.5) | 1 (0.003%) | 21 (0.003%) | N/A |
| Guillain-Barré syndrome | 1 (0.01%) | 56 (0.02%) | N/A | 1 (0.003%) | 5 (0.001%) | N/A |
| Multiple  sclerosis | 1 (0.01%) | 166 (0.07%) | N/A | 10 (0.03%) | 127 (0.02%) | 1.4 (0.7-2.7) |
| Myasthenia gravis | 1 (0.01%) | 37 (0.02%) | N/A | 0 (0.00%) | 24 (0.004%) | N/A |
| Narcolepsy | 0 (0.00%) | 11 (0.005%) | N/A | 0 (0.00%) | 26 (0.004%) | N/A |
| 12 months | | | | | | |
| Alzheimer’s disease | 7 (0.09%) | 55 (0.02%) | 3.7 (1.7-8.0) * | 22 (0.06%) | 116 (0.02%) | 3.5 (2.2-5.5) * |
| Parkinson’s disease | 3 (0.04%) | 73 (0.03%) | 1.2 (0.4-3.8) | 23 (0.07%) | 163 (0.03%) | 2.6 (1.7-4.0) * |
| Ischemic stroke | 146 (1.82%) | 7,002 (3.04%) | 0.6 (0.5-0.7) | 135 (0.38%) | 910 (0.14%) | 2.7 (2.3-3.2) * |
| Intracerebral hemorrhage | 11 (0.14%) | 311 (0.14%) | 1.0 (0.6-1.9) | 5 (0.008%) | 19 (0.003%) | 4.8 (1.8-12.9) * |
| Subarachnoid  hemorrhage | 7 (0.09%) | 260 (0.11%) | 0.8 (0.4-1.6) | 3 (0.01%) | 29 (0.004%) | N/A |
| Guillain-Barré syndrome | 1 (0.01%) | 57 (0.02%) | N/A | 1 (0.003%) | 7 (0.001%) | N/A |
| Multiple sclerosis | 1 (0.01%) | 184 (0.08%) | N/A | 13 (0.04%) | 149 (0.02%) | 1.6 (0.9-2.8) |
| Myasthenia gravis | 1 (0.01%) | 41 (0.02%) | N/A | 0 (0.00%) | 30 (0.005%) | N/A |
| Narcolepsy | 0 (0.00%) | 13 (0.006%) | N/A | 0 (0.00%) | 28 (0.004%) | N/A |

Statistical analyses were only conducted for diseases with ≥ 4 cases in each group.

* Statistically significant RR (p<0.05)

**^♦^** Excluding inpatient cases of Alzheimer’s and Parkinson’s disease the first three months after hospitalization with COVID-19

**5. Supplementary table 5 (S5)**

Relative risk of neurologic immune-mediated, neurodegenerative, and cerebrovascular disorders in COVID-19 positive compared to influenza positive individuals.

|  | COVID-19  positive  (n=43,262) | Influenza  positive  (n=8,102) | RR (95%CI) | COVID-19  positive  (n=43,262) | Influenza  positive  (n=8,102) | RR (95%CI) |
| --- | --- | --- | --- | --- | --- | --- |
| 1 month (n,%) | | | | **3 months (n,%)** | | |
| Alzheimer’s disease^♦^ | - | - | - | - | - | - |
| Parkinson’s disease^♦^ | - | - | - | - | - | - |
| Ischemic stroke | 117 (0.27%) | 26 (0.32%) | 0.8 (0.6-1.3) | 178 (0.41%) | 34 (0.42%) | 1.0 (0.7-1.4) |
| Intracerebral hemorrhage | 7 (0.02%) | 0 (0.00%) | N/A | 10 (0.02%) | 0 (0.00%) | N/A |
| Subarachnoid  hemorrhage | 4 (0.01%) | 0 (0.00%) | N/A | 5 (0.01%) | 0 (0.00%) | N/A |
| Guillain-Barré syndrome | 1 (0.00%) | 2 (0.02%) | N/A | 1 (0.00%) | 2 (0.02%) | N/A |
| Multiple sclerosis | 4 (0.01%) | 0 (0.00%) | N/A | 6 (0.01%) | 1 (0.01%) | N/A |
| Myasthenia gravis | 1 (0.00%) | 0 (0.00%) | N/A | 1 (0.00%) | 0 (0.00%) | N/A |
| Narcolepsy | 0 (0.00%) | 0 (0.00%) | N/A | 0 (0.00%) | 0 (0.00%) | N/A |
| 6 months (n,%) | | | | **12 months (n,%)** | | |
| Alzheimer’s disease | 21 (0.05%) | 2 (0.02%) | N/A | 29 (0.07%) | 5 (0.06%) | 1.1 (0.4-2.8) |
| Parkinson’s disease | 18 (0.04%) | 2 (0.02%) | N/A | 24 (0.06%) | 6 (0.07%) | 0.7 (0.3-1.8) |
| Ischemic stroke | 225 (0.52%) | 42 (0.52%) | 1.0 (0.7-1.4) | 279 (0.64%) | 65 (0.80%) | 0.8 (0.6-1.1) |
| Intracerebral hemorrhage | 13 (0.03%) | 0 (0.00%) | N/A | 16 (0.04%) | 1 (0.01%) | N/A |
| Subarachnoid  hemorrhage | 6 (0.01%) | 0 (0.00%) | N/A | 10 (0.02%) | 2 (0.02%) | N/A |
| Guillain-Barré syndrome | 2 (0.00%) | 2 (0.02%) | N/A | 2 (0.00%) | 2 (0.02%) | N/A |
| Multiple sclerosis | 11 (0.03%) | 2 (0.02%) | N/A | 14 (0.03%) | 3 (0.04%) | N/A |
| Myasthenia gravis | 1 (0.00%) | 0 (0.00%) | N/A | 1 (0.00%) | 0 (0.00%) | N/A |
| Narcolepsy | 0 (0.00%) | 0 (0.00%) | N/A | 0 (0.00%) | 0 (0.00%) | N/A |

Statistical analyses were only conducted for diseases with >3 cases in each group.

* Statistically significant RR (p<0.05)

**^♦^** Excluding inpatient cases of Alzheimer’s and Parkinson’s disease the first three months after hospitalization.

**6. Supplementary table 6 (S6)**

Relative risk of of neurodegenerative, cerebrovascular, and immune-mediated disorders across all groups after

removal of individuals with significative risk factors.

| After removal of individuals with cerebrovascular risk factors | | | | | | | | | | | | | | | | | | |  |
| --- | --- | --- | --- | --- | --- | --- | --- | --- | --- | --- | --- | --- | --- | --- | --- | --- | --- | --- | --- |
|  | Covid + | Influenza | RR (95%CI) | Covid + inpt | Influenza  inpt | RR  (95%CI) | Covid + inpt | Pneumonia | RR  (95% C.I.) | Covid + inpt | Covid - inpt | RR (95% C.I.) | Covid + outpt | Covid - outpt | RR (95% C.I.) | Covid + | Covid  - | RR (95% C.I.) |  |
| 1 month | | | | | | | | | | | | | | | | | | | |
| AD^♦^ | - | - | - | - | - | - | - | - | - | - | - | - | 3 | 22 | N/A | - | - | - |  |
| IS | 56 | 9 | 0.9  (0.5-1.9) | 29 | 6 | 3.4  (1.4-8.2)* | 26 | 2 | N/A | 32 | 1960 | 0.6(0.4-0.9) | 10 | 191 | 1.0  (0.5-1.9) | 42 | 2157 | 0.4  (0.3-0.5) |  |
| ICH | 2 | 0 | N/A | 1 | 0 | N/A | 1 | 0 | N/A | 1 | 92 | N/A | 0 | 8 | N/A | 1 | 100 | 0.2  (0.03-1.4) |  |
| SAH | 3 | 0 | N/A | 1 | 0 | N/A | 1 | 0 | N/A | 1 | 87 | N/A | 1 | 2 | N/A | 2 | 89 | 0.5 (0.1-1.8) |  |
| 3 months | | | | | | | | | | | | | | | | | | | |
| AD^♦^ | - | - | - | - | - |  | - | - | - | - | - | - | 6 | 36 | 3.1  (1.3-7.4)* | - | - | - |  |
| IS | 86 | 13 | 1.0  (0.6-1.8) | 39 | 9 | 3.0  (1.5-6.3)* | 37 | 3 | N/A | 42 | 2128 | 0.7  (0.5-1.0) | 22 | 233 | 1.8  (1.5-2.8)* | 64 | 2372 | 0.5  (0.4-0.7) |  |
| ICH | 2 | 0 | N/A | 2 | 0 | N/A | 2 | 0 | N/A | 2 | 103 | N/A | 0 | 10 | N/A | 2 | 113 | 0.4 |  |
| SAH | 4 | 0 | N/A | 2 | 0 | N/A | 2 | 0 | N/A | 2 | 100 | N/A | 1 | 3 | N/A | 3 | 103 | 0.6 |  |
| 6 months | | | | | | | | | | | | | | | | | | | |
| AD | 17 | 2 | N/A | 1 | 1 | N/A | 1 | 0 | N/A | 1 | 12 | N/A | 7 | 51 | 2.6  (1.2-5.7)* | 8 | 64 | 2.5  (1.2-5.2)* |  |
| IS | 110 | 14 | 1.2  (0.7-2.1) | 45 | 9 | 3.5  (1.7-7.2)* | 44 | 3 | N/A | 48 | 2265 | 0.8  (0.6-1.3) | 34 | 293 | 2.2  (1.5-3.1)* | 82 | 2573 | 0.6  (0.5-0.8) |  |
| ICH | 2 | 0 | N/A | 2 | 0 | N/A | 2 | 0 | N/A | 2 | 112 | N/A | 0 | 12 | N/A | 2 | 124 | N/A |  |
| SAH | 7 | 0 | N/A | 4 | 0 | N/A | 4 | 0 | N/A | 4 | 107 | 1.4  (0.5-3.7) | 2 | 5 | N/A | 6 | 112 | 1.1  (0.5-2.4) |  |
| 12 months | | | | | | | | | | | | | | | | | | | |
| AD | 19 | 4 | 0.7  (0.2-2.1) | 1 | 3 | N/A | 1 | 0 | N/A | 1 | 22 | N/A | 8 | 68 | 2.2  (1.0-4.6) | 9 | 55 | 3.3  (1.6-6.7)* |  |
| IS | 132 | 22 | 0.9  (0.6-1.4) | 51 | 13 | 2.8  (1.5-5.0)* | 51 | 5 | 0.6(0.2-1.5) | 54 | 2355 | 0.8  (0.6-1.1) | 44 | 398 | 2.1  (1.5-2.8)* | 98 | 2777 | 0.7  (0.6-0.9) |  |
| ICH | 4 | 2 | N/A | 2 | 0 | N/A | 2 | 0 | N/A | 2 | 123 | N/A | 1 | 19 | N/A | 3 | 142 | N/A |  |
| SAH | 10 | 1 | N/A | 4 | 1 | N/A | 4 | 0 | N/A | 4 | 116 | 1.3  (0.5-3.4) | 4 | 7 | 10.8  (3.1-36.7) * | 8 | 123 | 1.3  (0.6-2.7) |  |
| After removal of individuals with delirium | | | | | | | | | | | | | | | | | | |  |
| 1 month | | | | | | | | | | | | | | | | | | |  |
| AD | - | - | - | - | - | - | - | - | - | - | - | - | 7 | 38 | 3.4  (1.5-7.6)* | - | - | - |  |
| 3 months | | | | | | | | | | | | | | | | | | |  |
| AD | - | - | - | - | - | - | - | - | - | - | - | - | 12 | 63 | 3.5  (1.9-6.5)* | - | - | - |  |
| 6 months | | | | | | | | | | | | | | | | | | |  |
| AD | 21 | 0 | N/A | 4 | 1 | N/A | - | - | - | 4 | 34 | 3.4  (1.2-9.6)* | 17 | 85 | 3.7  (2.2-6.2)* | 21 | 119 | 3.6  (2.2-5.7)* |  |
| 12 months | | | | | | | | | | | | | | | | | | |  |
| AD | 28 | 2 | N/A | 7 | 3 | N/A | - | - | - | 7 | 55 | 3.7  (1.7-8.1)* | 21 | 117 | 3.3  (2.1-5.2)* | 28 | 172 | 3.3  (2.2-4.9)* |  |

| After removal of individuals with rheumatoid arthritis in COVID-19 compared to influenza | | | | | | | | | | | | |
| --- | --- | --- | --- | --- | --- | --- | --- | --- | --- | --- | --- | --- |
|  | Covid + | Influenza | RR (95%CI) | Covid + | Influenza | RR (95%CI) | Covid + | Influenza | RR (95%CI) | Covid + | Influenza | RR (95%CI) |
| 1 month | | | | **3 months** | | | **6 months** | | | **12 months** | | |
| GBS | 0 | 2 | N/A | 1 | 2 | N/A | 1 | 2 | N/A | 1 | 2 | N/A |
| MS | 3 | 0 | N/A | 5 | 1 | N/A | 7 | 2 | N/A | 8 | 3 | N/A |
| MG | 1 | 0 | N/A | 1 | 0 | N/A | 1 | 0 | N/A | 1 | 0 | N/A |
| N | 0 | 0 | N/A | 0 | 0 | N/A | 1 | 0 | N/A | 2 | 0 | N/A |

Statistical analyses were only conducted for diseases with ≥ 4 cases in each group.

* Statistically significant RR (p<0.05)

**^♦^** Excluding inpatient cases of Alzheimer’s disease the first three months after hospitalization.

AD = Alzheimer’s disease, IS = ischemic stroke, ICH = intracerebral hemorrhage, SAH = subarachnoid hemorrhage, GBS = Guillain-Barré syndrome, MS = multiple sclerosis,

N = narcolepsy.

**7. Supplementary table 7 (S7)**

Comparison of the incidence or prevalence of neurologic disorders in the COVID-19 negative population to the background population

|  | Incidence or prevalence of neurologic disorders in COVID-19 negative individuals | Previously published incidences and prevalences |
| --- | --- | --- |
| Dementia of any type (≥ 65 years) | 5 pr 1,000 pr year | 5 pr 1,000 pr year^a^ |
| Parkinson’s disease (≥ 18 years) | 0.3% | 0.3%^b^ |
| Ischemic stroke | 7 pr 1,000 pr year | 2.1 pr 100,000 pr year^1^ |
| Subarachnoid hemorrhage | 33 pr 100,000 pr year | 9 pr 100,000 pr year |
| Intracerebral hemorrhage (≥ 75 years) | 124 pr 100,000 pr year | 88-176 pr 100,000c pr year^2^ |
| Multiple sclerosis | 20 pr 100,000 pr year | 12 pr 100,000 pr year^3^ |
| Guillain-Barre syndrome | 4.8 pr 100,000 pr year | 1.5 pr 100,000 pr year^4^ |
| Myasthenia Gravis | 8 pr 100,000 pr year | Unknown in Denmark^p^ |
| Narcolepsy | 5 pr 100,000 pr year | 5 pr 100,000^p^ pr year^p^ |

^a^While there are no recent reports of the prevalence of specifically Alzheimer’s disease in Denmark^5^,The National Danish Dementia Research Center recently reported that in Denmark 8,000 individuals aged ≥ 65 years develop dementia per year (5 pr 1,000 per year)^6^.

^b^The prevalence of Parkinson’s disease in industrialized countries is estimated to be 0.3% in the general population.^7^  Furthermore, according to the Danish Society for Movement Disorders,^8^ the prevalence of Parkinson’s disease among Danish people between 65-75 years is 0.5-1%

^J^Third, a long-term study of 10,333 participants of 99% European ancestry reported a yearly incidence of 88 -176 intracerebral hemorrhages per 100,000 individuals ≥ 75 years^9^.

^p^Myasthenia Gravis and narcolepsy are both relatively rare and the incidence rates in Denmark are not known, to our knowledge. The incidence of narcolepsy in the COVID-19 population was, however, roughly in line with recent reports in another high-income country (United States).^10^

**References**

1. Dansk Apopleksiregister [online]. Available at: <https://www.sundhed.dk/content/cms/69/4669_dap_aarsrapport-2020_240621.pdf>. Accessed February 28, 2022.

2. Markus Harboe Olsen AL-C, Søren Bache, Vagn Eskesen, Kirsten Møller. Aneurismal subaraknoidal blødning.

3. Multipel Sklerose [online]. Available at: <https://sundhedsdatastyrelsen.dk/da/tal-og-analyser/analyser-og-rapporter/sygdomme-og-behandlinger/multipel_sklerose>. Accessed February 28, 2022.

4. Guillain-Barre Syndrom [online]. Available at: m<https://neuro.dk/wordpress/nnbv/guillain-barre-syndrom/>. Accessed February 28, 2022.

5. Nielsen H, Lolk A, Andersen K, Andersen J, Kragh-Sorensen P. Characteristics of elderly who develop Alzheimer's disease during the next two years-a neuropsychological study using CAMCOG. The Odense Study. Int J Geriatr Psychiatry 1999;14:957-963.

6. Forekomst af demens i Danmark [online]. Available at: <https://videnscenterfordemens.dk/da/forekomst-af-demens-i-danmark>. Accessed February 28, 2022.

7. von Campenhausen S, Bornschein B, Wick R, Botzel K, Sampaio C, Poewe W, et al. Prevalence and incidence of Parkinson's disease in Europe. Eur Neuropsychopharmacol 2005;15:473-490.

8. Parkinsons Sygdom [online]. Available at: <https://danmodis.dk/parkinsons-sygdom/>. Accessed February 28, 2022.

9. Lioutas VA, Beiser AS, Aparicio HJ, Himali JJ, Selim MH, Romero JR, et al. Assessment of Incidence and Risk Factors of Intracerebral Hemorrhage Among Participants in the Framingham Heart Study Between 1948 and 2016. JAMA Neurol 2020;77:1252-1260.

10. Scheer D, Schwartz SW, Parr M, Zgibor J, Sanchez-Anguiano A, Rajaram L. Prevalence and incidence of narcolepsy in a US health care claims database, 2008-2010. Sleep 2019;42.
